# Food Antimicrobials Nanocarriers

**DOI:** 10.1155/2014/837215

**Published:** 2014-06-03

**Authors:** Adriana Blanco-Padilla, Karen M. Soto, Montserrat Hernández Iturriaga, Sandra Mendoza

**Affiliations:** Departamento de Investigación y Posgrado en Alimentos, Facultad de Química, Universidad Autónoma de Querétaro, Cerro de las Campanas s/n, Querétaro, QRO 76010, Mexico

## Abstract

Natural food antimicrobials are bioactive compounds that inhibit the growth of microorganisms involved in food spoilage or food-borne illness. However, stability issues result in degradation and loss of antimicrobial activity. Nanoencapsulation allows protection of antimicrobial food agents from unfavorable environmental conditions and incompatibilities. Encapsulation of food antimicrobials control delivery increasing the concentration of the antimicrobials in specific areas and the improvement of passive cellular absorption mechanisms resulted in higher antimicrobial activity. This paper reviews the present state of the art of the nanostructures used as food antimicrobial carriers including nanoemulsions, nanoliposomes, nanoparticles, and nanofibers.

## 1. Introduction

Actually the consumer demand for safe natural minimally processed food has forced the food industry either to reduce the amount of synthetic antimicrobial substances or to replace them with natural alternatives. However, many of these natural compounds are not as effective as the synthetic additives, more expensive, or can negatively interact with food components. In this perspective, encapsulation procedures provide an effective protection of antimicrobial compounds against chemical reactions and undesirable interactions with other components in food, improve solubility, diminish migration, and preserve the bioactive compounds stability during food processing and storage. Furthermore, encapsulation of bioactives compounds controls delivery and improves absorption and bioavailability [1–3].

While microencapsulation systems may guarantee protection of antimicrobial compounds against degradation or evaporation, the high surface area to volume ratio of the nanoencapsulation systems (systems in the nanometer scale, smaller than 100 nm) can increase the concentration of the antimicrobials in specific food areas where microorganisms are preferably located [4] and improve passive cellular absorption mechanisms that could lead to higher antimicrobial activity. Moreover, the nanoencapsulation processes are helpful to incorporate antimicrobial agents in material used for food packaging [5]. An antimicrobial delivery system is designed to release the active at a particular site of action that can be on the surface or inside the microbial cell. Release can be controlled according to the environment surrounding the system (pH, temperature, ionic environment, or enzymatic activity). The nanoencapsulated antimicrobial may observe initially less activity compared with the nonencapsulated compound; however, the antimicrobial activity of the encapsulated compound lasts much longer [4]. The use of nanocarriers can modulate the release of antimicrobials, protecting them from adverse conditions, improving their stability, and directing them to the site of action, thereby decreasing the amount required to observe an antimicrobial effect. Each of the different encapsulation systems has advantages and disadvantages. In general, the nanoencapsulation systems have excellent sustained-release properties, subcellular size, and biocompatibility with tissue and cells, allow alterations in the bioavailability of drugs, and improve the pharmacokinetic profile of numerous actives [6]. Additionally, the encapsulation of antimicrobial compounds reduced their toxicity, the resistance is overcome, and the cost of using them is decreased because a less amount of the active is required [7]. Limitations of all nanoencapsulation systems for their use in food industry are related to their high production costs and lack of allowed materials [8].  Table 1 summarizes some advantages and disadvantages of the nanocarrier systems described in this review.

Many compounds have been encapsulated; some of them are antioxidants [9], flavors [10], and antimicrobial compounds [11]. This review focuses on the nanoencapsulation systems for food antimicrobials, discussing their variations, developments, and trends.

## 2. Nanoemulsions

**Figure 1:**
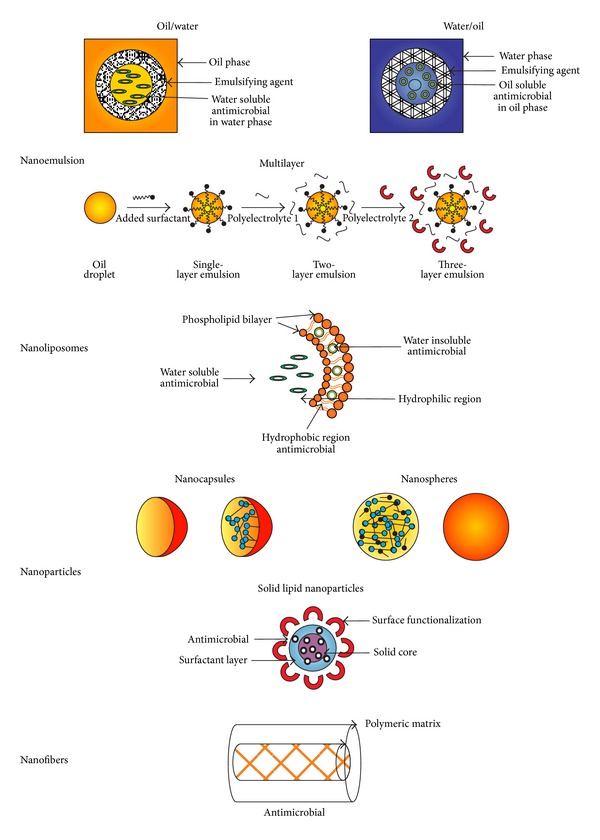
Structures of nanoscale delivery systems used to entrap food antimicrobials.

Nanoemulsions are stable colloidal systems within nanometric size (≤100 nm) formed by dispersing one liquid in another immiscible liquid using suitable emulsifiers (Figure 1) [12]. Compared with microemulsions, nanoemulsions are optically transparent, demonstrating better shelf stability, and the droplet size distribution remains after water dilution [13].

Nanoemulsions can be prepared with different materials depending on the desire structure and functionality by using high-energy methods (high-pressure homogenization, microfluidization, and ultrasonication) and low-energy methods (solvent diffusion). High-energy methods produce intense disruptive forces minimizing droplet size to form emulsions, while low energy methods promote spontaneous emulsification by mixing all the emulsion ingredients [12, 14, 15]. Among the most used nanoemulsions are (1) the oil in water (O/W) where the oil droplets are dispersed in the aqueous phase and the interphase is stabilized by emulsifiers; (2) the multiple emulsions oil-in-water-in-oil (O/W/O) and water-in-oil-in-water (W/O/W), where, for example, nanometer size water droplets contained within large oil droplets are dispersed within an aqueous phase (W/O/W); and (3) the multilayer emulsions which consist of oil droplets surrounded by nanometric size layers of different polyelectrolytes [16].

O/W nanoemulsions can encapsulate and deliver poorly water-soluble food antimicrobials improving physical stability of the active compound and increasing its active distribution in food matrices. The nanometric droplets size has the advantage of increasing interactions of the active compound with the cell membrane of bacteria, affecting the stability of the lipid membrane and resulting in leakage of bacteria intracellular constituents. This nonspecific action mechanism decreases the development of resistant microbial strains.

Ideally, an optimal delivery system for antimicrobial compounds that could have application in food industry would enhance the mass transfer rates to the sites of action, in order to maximize the antimicrobial activity and to use concentrations which are low enough to minimally alter the quality of the product, but are sufficient to inhibit microbial growth within the limits of food regulations [17].

Some food antimicrobial agents have been encapsulated in nanoemulsions (Table 2). Essential oils are generally recognized as safe (GRAS) food additives according to the United States Food and Drug Administration (FDA) and display activities against human pathogenic and food spoilage microorganisms; however water solubility constrains, evaporation, and sensory properties have limited their incorporation in food products [18, 19]. Therefore, encapsulation of essential oils at the nanoscale represents an available and efficient approach to increasing their physical stability and reducing the mass transfer resistances of the active molecules to the sites of action.

**Figure 2:**
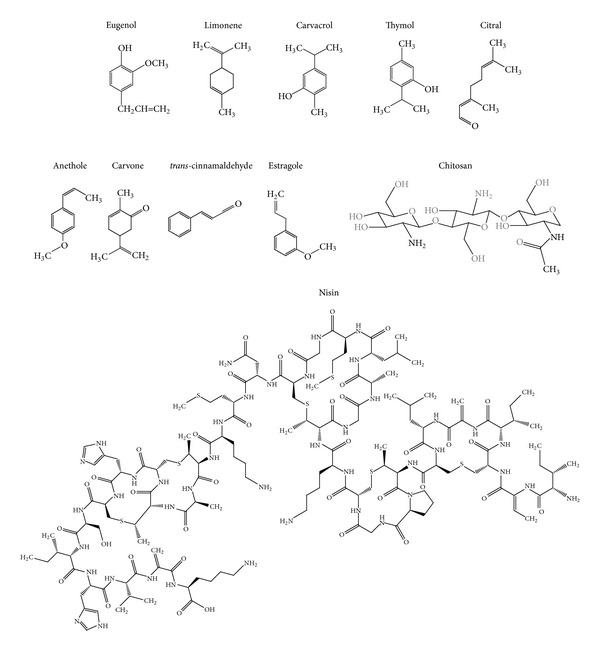
Structures of natural food antimicrobials.

Eugenol (Figure 2) was incorporated in O/W nanoemulsion using sesame oil, Tween 80, and water by ultrasound cavitation method. The nanoemulsion with 0.003% of eugenol was stable for more than one month and exhibited antibacterial activity against* Staphylococcus aureus*, after 120 minutes the population of the microorganism was reduced 3 log (CFU/mL) due to membrane permeability changes [15]. Terjung et al. [20] developed nanoemulsions containing carvacrol (Figure 2) and eugenol with triacylglyceride (Miglyol 812N) or Tween 80 by high-pressure homogenization and ultrasonication. The antimicrobial activity of emulsions was tested against* Escherichia coli* C 600 and* Listeria innocua*. Carvacrol emulsions with a mean oil droplet size of 3000 nm at a concentration of 800 ppm completely inhibited* L. innocua*, while for 80 nm emulsions, only a delay of growth was observed. In this case, the authors attribute the fact that antimicrobial nanoemulsions were less active than macroemulsions due to an increased sequestering of antimicrobials in emulsion interfaces and a decreased solubilization in excess Tween 80 micelles [20].

Carvacrol, limonene, and cinnamaldehyde were encapsulated in the sunflower oil based nanoemulsions obtained by high-pressure homogenization and stabilized by different emulsifiers (Figure 2). The antimicrobial activity was measured against* Escherichia coli*,* Lactobacillus delbrueckii*, and* Saccharomyces cerevisiae*. The antimicrobial activity was dependent on the compound concentration in the aqueous phase which was governed by the emulsifier capability to solubilize them. Emulsifiers such as sugar esters and glycerol monooleate solubilize the essential oil in the aqueous phase at high concentrations resulting in a high antimicrobial activity. Carvacrol emulsion achieved complete inactivation of* E. coli* and* L. delbrueckii* after two hours while reduced the population of* S. cerevisiae* (2log) after two hours. Complete inactivation of* S. cerevisiae* was reached after 24 hours. Limonene and cinnamaldehyde emulsions exhibited lower antimicrobial activity than carvacrol; the more concentrated essential oil nanoemulsions demonstrated complete inactivation of* E. coli*,* L. delbrueckii*, and* S. cerevisiae* after 24 h. Due to a high availability of the compound a significantly enhanced bactericidal effect over shorter time scales compared with nonencapsulated essential oil was observed. Emulsifiers that slightly solubilized the essential oil in the aqueous phase (lecithin and pea proteins) promote bacteriostatic action [21].

Basil oil (*Ocimum basilicum*) containing 88% of estragole (Figure 2) was encapsulated in a nanoemulsion formulated with Tween 80 and water by ultrasonic emulsification method. The nanoemulsion showed antibacterial activity against* E. coli* even after being diluted. For example, 10-fold and 100-fold dilutions inactivated completely* E. coli* after 45 minutes while the 1000-fold dilution achieved a reduction of 40% after 60 minutes. Fluorescence microscopy and FT-IR results showed that nanoemulsion promotes bacterial cell membrane alterations [22].

Lemongrass oil (LO) has been encapsulated in a carnauba-shellac wax (CSW) based nanoemulsion by high pressure homogenization [19, 23] and alginate nanoemulsions by ultrasonication and microfluidization [18]. The lemongrass oil loaded CSW-based nanoemulsions decreased by 8.18 log CFU/g the total population of* E. coli* O157:H7 and* L. monocytogenes* after 2 hr. As edible coating, after five months of storage, the unloaded and LO-loaded CSW nanoemulsions applied to apples allowed a decrease of 0.8 and 1.4 log CFU of aerobic bacteria, respectively. The coatings inhibited the development of yeast and molds. Moreover, the coating inhibited the growth of* Salmonella* typhimurium and* E. coli* O157:H7 on apples and plums, respectively. Additionally, the application of nanoemulsions preserved various physicochemical qualities of fruits [19, 23].

The LO-alginate nanoemulsions demonstrated antibacterial effect against* E. coli*; however the biological activity was dependent on the nanoemulsion production process. While microfluidization enhanced antimicrobial activity, ultrasounds diminished the activity.

Joe et al. [24] developed a sunflower oil-surfactin-based O/W nanoemulsion. The synthetic surfactants were replaced by surfactin, a cyclic lipopeptide antibiotic biosurfactant produced by* B. subtilis*. The nanoemulsion demonstrated high antibacterial activity against* S.* Typhi,* L. monocytogenes*, and* S. aureus* compared with streptomycin, positive control, at 100 mg/L; high fungicidal activity against* Rhizopus nigricans*,* Aspergillus niger*, and* Penicillium* sp. compared with sodium benzoate, positive control, and good sporicidal activity against* Bacillus cereus* and* Bacillus circulans* (3-fold than positive control). When the sunflower oil-surfactin nanoemulsion was applied to food products such as raw chicken, apple juice, milk, and mixed vegetable, a reduction in the native cultivable bacterial and fungal populations was observed.

Bovine lactoferrin is an iron-binding protein that strongly inhibits growth or kills iron-dependent pathogenic bacteria. It was entrapped within W/O/W multiple nanoemulsions with lecithin and poloxamers by homogenization. Both free and encapsulated lactoferrin showed a minimum inhibitory concentration (MIC) of 2000 mg/mL for* S. aureus* and* L. innocua* and 200 mg/mL for* Candida albicans*. Although an improvement in antibacterial activity was not observed upon encapsulation, the activity remained and these multiple nanoemulsions can be employed to formulate oral elixir and beverages [25].

## 3. Nanoparticles

The term nanoparticle is used for both nanospheres and nanocapsules (Figure 1). A nanosphere is a polymeric matrix where the actives may be absorbed at the sphere surface or encapsulated within the particle. A nanocapsule is a vesicular system in which the active is confined to an inner liquid core [6, 26]. The functional performance of nanoparticle-based delivery systems depends on their physicochemical properties such as size, morphology, charge, and physical state [27, 28].

Salting out, spontaneous emulsification/diffusion, solvent evaporation, polymerization, nanoprecipitation, and electrospraying are examples of suitable methods to produce nanoparticles with food applications [16].

Solid lipid nanoparticles (SLN) are particles consisting of a matrix solid lipid shell (Figure 1). Among the advantages compared to nanoemulsions and liposomes, SLN do not require organic solvents for their preparation and exhibit higher encapsulation efficiencies as well as longer times for release. For food processing, hot and cold homogenizations are recommended methods [13].

Several natural compounds with antimicrobial and antifungal activities have been encapsulated in nanoparticles (Table 2). Gomes et al. [29] developed spherical poly(DL-lactide-co-glycolide) (PLGA) nanoparticles loaded with eugenol and* trans*-cinnamaldehyde (Figure 2) by the emulsion-evaporation method using poly(vinyl alcohol) (PVA) as a surfactant. The loaded nanoparticles effectively inhibit the growth of* Listeria* spp. and* Salmonella* spp. with minimum inhibitory concentrations (MIC) ranging from 10 to 20 mg/mL, respectively. The nanoencapsulation improved the water solubility of eugenol and* trans*-cinnamaldehyde and demonstrated a sustained release with continuous migration of the antimicrobial during 72 hr.

Esfandyari-Manes et al. [30] prepared PLGA nanospheres loaded with anethole and carvone (Figure 2) by both emulsification solvent evaporation and nanoprecipitation methods. Carvone-loaded nanoparticles demonstrated a MIC against* S. aureus* and* E. coli* bacteria of 182 and 374 mg/mL, respectively, while anethole-loaded nanoparticles exhibited a MIC of 227 mg/mL against* S.* Typhi. Unloaded nanoparticles and DMSO was used as control and did not have any antimicrobial effect. Keawchaoon and Yoksan [31] produced spherical carvacrol-loaded chitosan SLN by a two-step method, O/W emulsion followed by ionic gelation of chitosan. These nanoparticles showed antimicrobial activity against* S. aureus*,* B. cereus*, and* E. coli* with a MIC of 0.257 mg/mL and minimum bactericidal concentration (MBC) of 4.113, 2.056, and 8.225 mg/mL, respectively. Unloaded chitosan nanoparticles did not show antimicrobial and antibacterial effect. In vitro release experiments reveled that after 60 days, carvacrol was released at 52.6% in acetate buffer solution at pH = 3, and 22.5% and 33.1% in phosphate buffer solutions adjusted at pH values of  7 and 11, respectively.

Thymol and carvacrol (Figure 2) were encapsulated in zein nanospheres by the liquid-liquid dispersion method. Both nanoparticles significantly decreased the concentration of nonpathogenic* E. coli* by 0.8–1.8log CFU/mL compared to the control that contained the same amount of* E. coli* without addition of antimicrobial agents [32]. Zhang et al. [33] improve the properties of the thymol-loaded zein nanoparticles by using sodium caseinate (SC) and chitosan hydrochloride (CHC) as stabilizers. Loaded SC and CHC-SC stabilized zein nanospheres at thymol concentration of 0.052 and 0.020 mg/mL, respectively, were tested against* E. coli*,* P. aeruginosa*,* C. albicans*, and* S. aureus*. Loaded nanoparticles showed more efficient growth inhibition of* S. aureus *than free thymol. Li et al. [34] obtained thymol-loaded core/shell zein/SC nanoparticles by an antisolvent procedure, which consist on directly pouring sodium caseinate (SC) into zein solutions.* E. coli* and* S. enterica* were sensitive to the thymol loaded zein/SC nanoparticles at thymol-to-zein ratios of 30–40%; moreover, the thymol-loaded nanoparticles decreased the population of* S. aureus* by 1–3 log cycles more than free thymol after 13 days of storage.

**Figure 3:**
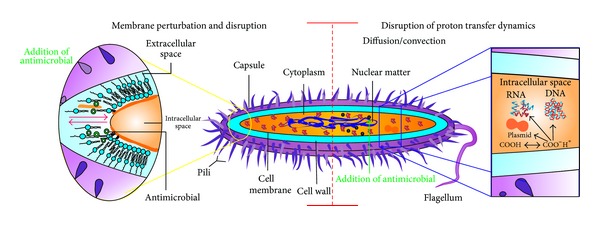
Mechanisms of action of antimicrobials.

Actually, antimicrobial peptides (AMPs) are alternatives to the use of antibiotics [35]. Nisin (Figure 2), a heat-stable peptide produced by* Lactococcus lactis* subsp.* lactis*, is the only antimicrobial bacteriocin with the status of GRAS approved by the FDA and it is the most utilized AMP in the food industry as food biopreservative [36]. Nisin offers effective control against the foodborne pathogens such as* L. monocytogenes*,* S. aureus*, and* B. cereus* [37]. This AMP kills susceptible bacteria through a multistep process that destabilizes the phospholipids bilayer of the cell and creates transient pores followed by leakage of the cellular materials, such as proteins and lipids [38]. Antimicrobials may act in two principal ways (Figure 3). Some of them, like nisin, can form pores in the membrane of sensitive cells; for this reason, the membrane loses its capability to act as a barrier, preventing exchange of material between the cell interior and exterior, leading to the efflux of cellular constituents and the collapse of proton-motive force [39]. Others may disrupt the membrane inserting them into the membrane structure changing the functionality. The interaction of nisin with food components (divalent cations, enzymes, and fat) promotes loss of antimicrobial activity.

Chitosan/carrageenan nanocapsules loaded with nisin were prepared by an ionic complexation method. Antimicrobial activity was evaluated against* Pseudomonas aeruginosa*,* S. enterica*,* Micrococcus luteus*, and* Enterobacter aerogenes*. The antibacterial effect in case of pure nisin lasted for 3 days and thereafter the bacterial growth commenced. The antibacterial effect of encapsulated nisin lasted for at least up to 20 days. In addition, the nisin nanocapsules showed antibacterial effect in tomato juice [10]. Xiao et al. [40] prepared nisin-loaded zein nanocapsules by spray drying at different temperatures. At 400 IU/mL, the encapsulated nisin showed slightly improved antibacterial activity against* L. monocytogenes* Scott A than free nisin in reduced fat milk.

Nisin was incorporated in SLN by high pressure homogenization. The platelet-shape nisin- loaded SLN exhibited antibacterial activity against* L. monocytogenes* and* Lactobacillus plantarum* for up to 20 and 15 days, respectively, compared to only one and three days, respectively, for free nisin [41].

Besides the antimicrobial agents that can be nanoencapsulated, there are some antimicrobial materials that are used to build the nanoparticles. That is the case of the zinc oxide (ZnO), currently listed as GRAS by the FDA and an important micronutrient for body growth and development. ZnO nanospheres prepared by hydrothermal synthesis were highly effective against* S.* Typhimurium and* S. aureus*. An edible film was prepared with ZnO nanoparticles and calcium alginate was used in a ready-to-eat-poultry meat challenge study, showing antimicrobial activity against the same pathogens by reducing 2 log CFU/mL the initial number of inoculated bacteria (10^6^-10^7^ CFU/mL) within 24 h. No living cells were detected after eight days [42].

## 4. Nanoliposomes

Nanoliposomes are nanometric spherical core shell structures, where hydrophobic hydrocarbon tails of phospholipids are associated into a bilayer and the polar head groups are directed to the aqueous phases of the inner and outer media (Figure 1). Liposomes structures can encapsulate, deliver, and release water-soluble, lipid-soluble, and amphiphilic materials. For food industry application, nanoliposomes can be produced by using natural sources, such as egg, soy, or milk that contained phospholipids with biological activity. Methods to produce nanoliposomes without employing toxic solvents are the microfluidization and heating [43].

The interaction of liposome structures with target cells may occur by adsorption onto the cell surface, fusion with the cell membrane, and release of active by micropinocytosis and due to a specific or nonspecific endocytosis [44].

Some food antimicrobials have been incorporated in nanoliposomes (Table 2). Nanoliposomes of nisin in soybean-lecithin were afforded by microfluidizer, a high-pressure homogenization method. Due to the amphiphilic character of nisin, it was encapsulated in both core and lamellar phases of nanoliposomes. Transmission electron microscopy experiments confirmed that nisin produced pores in the nanoliposomes. Not only the slow degradation of nanoliposomes but also pore formation by nisin played a role in controlled release of the bacteriocin. Further, the nisin nanoliposomes were embedded in a hydroxypropyl methylcellulose (HPMC) matrix to slowdown release of nisin. The HPMC film forming solution containing the nanoemulsion formulation (nisin-loaded nanoliposomes and free nisin) demonstrated better antimicrobial activity against* L. monocytogenes* compared with the nisin-loaded nanoliposomes, HPMC film solution and nisin-HPMC film solution. The nisin nanoemulsion HPMC film solution inhibited the development of* L. monocytogenes* during the experimental time frame (80 hr). The nisin-loaded nanoliposomes HPMC film solution inhibited the development of the microorganism before 10 hr; thereafter a growth was observed [45].

## 5. Nanofibers

Nanofibers are ultrathin structures with diameters below 100 nm and are produced mostly by electrospinning which is a process that produces continuous polymer fibers through the action of an external electric field imposed on a polymeric solution or melt. Materials such as proteins, carbohydrates, and lipids can be used. Recently, the electrospun nanofibers have drawn great interest to food industry because of their high surface area-to-volume ratios. This property makes the mats composed of electrospun fibers excellent candidates for various applications, like edible films and additive delivery systems.

Most of the nanofibers works are devoted to build active packing, but some examples can be found on electrospun fibers with antimicrobial properties (Table 2). Cationic cellulose derivatives showed good hydrophilicity, biodegradability, and antibacterial properties which allowed its use in textile, food, cosmetics, and pharmaceutical industries. Electrospun nanofibers were obtained by the polymeric mixture of Polyquaternium-4 cellulose (PQ-4), a hydroxyethyl cellulose diallyl dimethyl ammonium chloride copolymer, and PVA. The nanofibers showed effective antibacterial activities against* E. coli* and* S. aureus* [46].

Chitosan (Figure 2) is a polysaccharide typically produced by partial N-deacetylation of the natural polymer chitin. Chitosan derivatives with quaternary ammonium groups possess high efficacy against bacteria and fungi and the target site of these cationic polymers is the cytoplasmic membrane of bacterial cells [47].

Ignatova et al. [48] reported the preparation of electrospun nanofibers with quaternized chitosan (QCh) and PVA. These fibers showed good antibacterial activity against* S. aureus* and* E. coli*. Electrospun mats with QCh-poly(vinyl pyrrolidone) (PVP) nanofibers submitted to photocrosslinking also showed high antibacterial activity against* S. aureus*, inhibiting its growth after 30 min contact. 98.8% reduction of bacteria* E. coli* was observed after 90 min of contact [49].

Cellulose-based products can be applied to food products due to their edibility, biodegradability, and good carrier and antibacterial properties. For example, bacterial cellulose (BC) produced by* Acetobacter xylinum* is used in food processing because of its chemically pure form of cellulose, high water holding capacity and tensile strength, high fiber content, and low cost. A crosslinking technique was used to produce nanofibers based on BC, *ε*-polylysine, a natural peptide with antibacterial properties, and procyanidins as crosslinkers. The nanofibers exhibited growth inhibition of* E. coli* and* S. aureus*, showing inhibition halos of 2.2 and 1.8 mm, respectively [50]. A mixture of zein, a hydrophobic prolamine protein derived from corn, and chitosan was electrospun to afford water insoluble nanofibers with antibacterial properties against* S. aureus* at pH 4.6 [51].

## 6. Nanoscience in Food Industry

Nanotechnology is a multidisciplinary field that focuses on the understanding and development of materials based on nanoscale structures [52]. Its applications are not limited and can be applied to different areas like textiles and pharmaceutical and food industries [53]. Current application of nanotechnology in sectors such as food and agricultural industry is limited since toxicological and regulatory issues are a big concern [54]. However, nanotechnology is being used to improve the quality and safety of food through the encapsulation and protection of antimicrobials that are highly unstable in food [55].

Nanotechnology is not considered as an emerging science; Sanguansri and Augustin in 2006 published a review on the use of nanomaterials in the food industry, where a chronology is from 2000, the year in which Kraft Foods formed a consortium of 15 universities and government laboratories called Nanoteck, which is dedicated to the production of nanocapsules with different methods of elaboration [56]. Today many countries are moving into the area with nanotechnology applications in the food industry, among the countries with major publications in the field are USA, Spain, China, Germany, and England [57].

Nanoencapsulation is mainly applied in the pharmaceutical industry, but its use in the food industry has opened up space quickly. Among the main uses of nanoencapsulation in food is the development of antimicrobial active packaging. In a study by Global Trends and Forecasting, it is estimated that the market of nanoencapsulated food additives will grow at a CAGR of 6.2% from 2013 to 2018 and reach $26,208.3 million by 2018 [57, 58].

## 7. Concluding Remarks

Compared to micro-sized presentations, nanocarriers provide more surface area, enhance solubility, and improve bioavailability and targetability. However, the availability of materials recognized as GRASS to be used to produce the nanoencapsulating systems has limited the research in food areas. There is a need of information regarding the interaction of the nanosystems with food matrices, the mechanisms of the release of nanoencapsulated food components, and toxicological studies. To apply the nanocarriers in food industry, future research has to focus on the design of scalable methods and identification of low-cost ingredients. Antimicrobial food nanocarriers may be suitable for controlling spoilage and growth of pathogenic microorganisms in foodstuff.

## Figures and Tables

**Table 1:** Advantages and disadvantages of nanoencapsulated systems of food antimicrobials.

Delivery system	Physical morphology	Advantage	Disadvantage	Reference
Nanoemulsions	Liquid	Transparent/translucent systems suitable to use in beverages Carrier of hydrophobic compounds Rapid absorption Toxicological safe Good shelf stability	Rapid release Low stability in acidic condition Low-energy methods are limited in food sector	[13] [59]

Nanoparticles				
(a) Nanospheres	Solid	Large surface-to-volume ratios Controlled release of insoluble actives	Lack of stability of some actives High production costs	[60] [61]
(b) Nanocapsules	Solid	The use of natural polymers such as polysaccharide and proteins can increase bioavailability and biodegradability	Large dispersion of encapsulated actives A purification process is necessary after the synthesis of nanocapsules	[60] [61] [62] [63]
(c) Solid lipid nanoparticles	Solid	Increase the aqueous solubility of the compound Produce a prolonged release and decrease the toxic side effects of the compound Rapid formulation development	Recrystallization risk and low encapsulation load	[13] [64]

Liposomes	Liquid	Capability to either encapsulate water-soluble drugs in their cavity or to solubilize lipophilic drugs in their bilayer Targetability High stability of compounds in foodstuff with high water content Large-scale production	Rapid release Short shelf lives	[65] [66] [67]

Nanofibers	Solid	Large surface area and porosity Possibility of large-scale production Capability to carry heat sensitive compounds High gas permeability	Biopolymers solubility limits their use in electrospinning	[68] [69] [70]

**Table 2:** Examples of food antimicrobials encapsulated in nanoemulsions, nanospheres, nanocapsules, and nanoliposomes.

Antimicrobial	Carrier system	Material used	Encapsulation efficiency (%)	Main target microorganism	Reference
Eugenol	Nanoemulsion	Sesame oil Miglyol 812N	—	*Escherichia coli *O157:H7 *Listeria monocytogenes Escherichia coli C 600, Listeria innocua *	[15] [20]
	Nanospheres	PLGA	98	*Salmonella *spp. *Listeria *spp.	[29]

Carvacrol	Nanoemulsion	Miglyol 812N Sunflower oil	— —	*Escherichia coli C 600, Listeria innocua* *Lactobacillus delbrueckii Saccharomyces cerevisiae *	[20] [21]
	Nanospheres	Chitosan Zein	14–31 —	*Staphylococcus aureus Bacillus cereus* *Escherichia coli *	[31] [32]

Thymol	Nanospheres	Zein	—	*Staphylococcus aureus *	[34]

Carvone and anethole	Nanospheres	PLGA	14.73 and 12.32	*Salmonella* Typhi	[30]

Limonene	Nanoemulsion	Sunflower oil	—	*Lactobacillus delbrueckii Saccharomyces cerevisiae* *Escherichia coli *	[21]

Cinnamaldehyde	Nanospheres	PLGA	92	*Salmonella *spp. *Listeria *spp.	[29]
	Nanoemulsion	Sunflower oil	—	*Lactobacillus delbrueckii Saccharomyces cerevisiae* *Escherichia coli *	[21]

Basil oil	Nanoemulsion	Basil oil/water	—	*Escherichia coli *	[22]

Lemongrass oil	Nanoemulsion	Carnauba Lemongrass oil/alginate	—	*Escherichia coli* O157:H7 *Salmonella *Typhimurium Yeast	[18] [19] [23]

Bovine lactoferrin	Nanoemulsion	Lecithin and poloxamers	—	*Staphylococcus aureus Listeria innocua * *Candida albicans *	[25]

Nisin	Solid Lipid Nanoparticles	Imwitor 900	73.6	*Lactobacillus plantarum Listeria monocytogenes *	[41]
	Nanocapsules	Chitosan/carrageenan Zein	53–93.32 36.65–49.05	*Pseudomonas aeruginosa Salmonella enterica Micrococcus luteus* *Enterobacter aerogenes Listeria monocytogenes *	[10] [40]
	Nanoliposomes	Soy bean/lecithin	50	*Listeria monocytogenes *	[45]
